# Improving Total-Cholesterol/HDL-Cholesterol Ratio Results in an Endothelial Dysfunction Recovery in Peripheral Artery Disease Patients

**DOI:** 10.1155/2012/895326

**Published:** 2012-09-25

**Authors:** Silvia Bleda, Joaquín de Haro, César Varela, Leticia Esparza, Javier Rodriguez, Francisco Acin

**Affiliations:** Vascular Surgery and Angiology Department, Hospital Universitario Getafe, Ctra Toledo Km 12,500, 28905 Madrid, Spain

## Abstract

*Aims*. To evaluate the effects of variations of total-cholesterol/HDL-cholesterol ratio and the effects of the atorvastatin on endothelial function in peripheral artery disease (PAD). *Material and Methods*. A prospective, randomised controlled study was carried out in 150 PAD patients. Patients randomized to the control group (*n* = 75) were treated with antiplatelet drugs, angiotensin-converting-enzyme inhibitors and cardiovascular-risk-factor control. Experimental group (*n* = 75) also received treatment with atorvastatin for a month. It was determined baseline nitrite plasma levels and total-cholesterol/HDL-cholesterol ratio and after one month of treatment in both groups. It was also analysed the correlation between the gradient of nitrite levels and the differential of total-cholesterol/HDL ratio in treatment group. *Results*. After a month, a reduction in nitrite levels was detected in treatment group (11.88 ± 7.8 **μ**M versus 5.7 ± 1.8 **μ**M, *P* < 0.0001). It was shown a higher decrease in nitrite plasma levels in the atorvastatin group finding lower levels assessments (5.7 ± 1.8 **μ**M versus 13.1 ± 9.1 **μ**M, resp., *P* < 0.001). A significant reduction in total-cholesterol/HDL-cholesterol ratio was observed in statin group after treatment (*P* < 0.0001). A strong correlation was found between the gradient of nitrite levels and the differential of total-cholesterol/HDL-cholesterol ratio in atorvastatin group (*r* = 0.7; *P* < 0.001). *Conclusions*. Improvement of nitrite levels are associated with decreased total cholesterol/HDL ratio values in PAD patients treated with atorvastatin.

## 1. Introduction

Peripheral arterial disease (PAD), the clinical manifestation of atherosclerosis in the lower limbs, is caused by a systemic chronic inflammatory state that affects the entire vascular system [1, 2]. Endothelial damage plays a crucial role in the etiopathogenia of this disease [3].

Endothelial dysfunction is considered to be an early event of atherosclerosis, preceding evidence of atherosclerotic plaques on angiography or ultrasound scan. Such endothelial dysfunction has been attributed to an impairment in nitric oxide (NO) bioactivity, and an increase in the formation of reactive oxygen species (ROS) [4]. NO has several antiatherosclerotic effects, such as inhibition of monocyte migration, inhibition of smooth muscle cell proliferation, and inhibition of platelet aggregation [2–4]. 

Nitrites are the product of the oxidation of the NO derived from the endothelium. Under physiological conditions, 70%–90% of the nitrites in plasma stem from endothelial nitric oxide synthase (eNOS) activity. Patients with PAD have been shown to have increased plasma nitrite levels from the early stages of the disease though this condition is unrelated to the severity of PAD [3].

In turn, hypercholesterolemia is an independent well-established risk factor for the development of atherosclerosis [5]. Previous studies have demonstrated that hypercholesterolemia is associated with endothelial dysfunction [6–8]. It is known to reduce endothelial function either by decreasing the synthesis and release of endothelium-derived relaxing factors or by inactivating NO after its release from endothelial cells by its reactions with superoxide radicals [9]. 

Meanwhile, high-density lipoprotein (HDL) has antiatherosclerotic effects, including augmentation of ability of reverse cholesterol transport, anti-inflammatory activity, inhibition of low-density lipoprotein (LDL) cholesterol oxidation, endothelial cell apoptosis, platelet aggregation, expression of cellular adhesion molecules, and re-endo-vascularization [10, 11].

Plasma HDL levels have been inversely related to the incidence of atherothrombotic disease [12, 13]. Part of its atheroprotective effect has been associated to its role in preserving endothelial function [14, 15]. In fact, several studies have suggested that a decrease in total-cholesterol/HDL-cholesterol ratio is linked to a reduction in the risk of morbidity and mortality in cardiovascular diseases [16–18].

There is some evidence that cholesterol-lowering therapy improves endothelium-dependent vasodilation in patients with normal lipid profile [19, 20]. However, there is little information regarding the effects of changes in total-cholesterol/HDL-cholesterol ratio per se on endothelium function in subjects with PAD.

The purpose of this study was to evaluate the effects of variations of total-cholesterol/HDL-cholesterol ratio on endothelial function indirectly determined by means of measurement of nitrite plasma levels in subjects with PAD. We evaluated the effects of the 3-hydroxy-3-methyl-glutaryl-CoA reductase (HMG-CoA reductase) inhibitor atorvastatin on endothelial function in these subjects.

## 2. Material and Methods

A prospective, experimental, randomised controlled, and translational study was carried out. There were sequentially included and randomized 150 PAD patients with Fontaine stage II at the time of diagnosis, confirmed by means of haemodynamic study (Doppler) and treadmill stress tests. All the patients included have not undergone previous revascularization procedure and were not received treatment with statins or contraindications for their use. We also excluded all patients with coexistence of chronic inflammatory diseases or steroidal medication.

**Figure 1:**
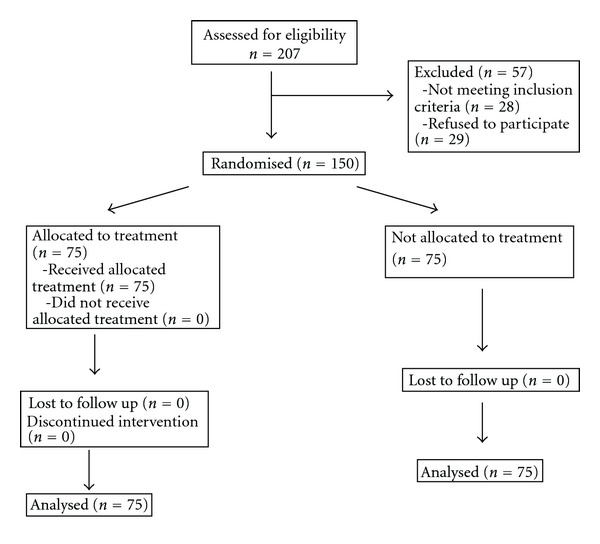
Method of randomization and allocation concealment.

The target patients who met the inclusion criteria were recruited and assessed in a screening visit at the outpatient clinic of the Angiology and Vascular Surgery Department of the Getafe University Hospital. When the criteria were met, and after signing the informed consent form, the patients were randomly allocated in a 1 : 1 ratio to the experimental intervention arm or the control group (Figure 1). A computer-generated random number sequence was used for randomization. The sequence was concealed until the study arm was assigned. There were three independent vascular surgeons investigators: one who recruited and evaluated the potential target patient in the screening visit and recorded cardiovascular risk factors, treatment, and general condition at the inclusion and after a month of the inclusion, blinded to the arm to which the patients were randomized; one who generated the allocation sequence; another who enrolled participants and assigned them to one of the study groups. Patients in the control group (*n* = 75) were prescribed treatment with aspirin 100 mg or clopidogrel 75 mg daily, if aspirin intolerant, angiotensin-converting-enzyme (ACE) inhibitors, and cardiovascular-risk-factors control. All patients in the experimental group (*n* = 75) also received atorvastatin 40 mg daily for a month.

Cardiovascular risk factors, treatment, and general condition were recorded on inclusion and after a month of treatment with statin. Ankle brachial index (ABI) was measured at rest as per the standard technique in the dorsalis pedis and posterior tibial arteries of both lower limbs [21]. Blood tests were performed at baseline and after one month of treatment with statin, which included basic clinical chemistry (glycemia, renal function, electrolytes, etc.) and lipid profile. Patients with plasma total cholesterol greater than 6.5 mmol L^−1^, LDL cholesterol greater than 3.2 mmol L^−1^ or triglycerides greater than 2.25 mmol L^−1^, or those on lipid-lowering treatment were considered to have dyslipidaemia [22]. Patients were considered to be hypertensive if they presented with systolic blood pressure greater than 140 mmHg and/or diastolic pressure greater than 90 mmHg and/or were on antihypertensive treatment for at least 1 year prior to inclusion in the study [23]. Patients were considered diabetic if they presented with baseline blood sugar greater than 120 g dL^−1^ or if they required treatment with hypoglycaemics [24]. Chronic renal failure was defined as serum creatinine greater than 1.5 mg dL^−1^ [25].

For the determination of plasma nitrite levels, the subjects came to the study having fasted for at least 12 hours and without having taken their usual medication during that period. Blood was drawn from an antecubital vein and centrifuged for 10 min at 800 g, with plasma then being stored at −4°C. Plasma nitrite concentrations were determined by colourimetric assay based on the Griess reaction [26]. This is a chemical reaction which uses sulfanilamide and N-(1-naphthyl) ethylenediamine dihydrochloride (NED) under acidic conditions (phosphoric acid). The system can detect NO_2_^−^ in a variety of biological and experimental fluids, the limit of detection being 2.5 mM (125 pmol). Each sample was analysed in triplicate, taking the mean of the three determinations. The blood tests were repeated in a control group of 10 patients to assess the reproducibility of the test, the coefficient of variation being less than 5%.

Cholesterol and triglyceride levels were measured by enzymatic techniques [27, 28]. HDL cholesterol was measured after precipitation of apoB-containing lipoproteins with polyanions [29] and VLDL cholesterol after separation of VLDL (*d* < 1.006 g · mL^−1^) by ultracentrifugation [30]. The LDL cholesterol was calculated by subtracting VLDL and HDL cholesterol from total cholesterol.

The laboratory data were determined anonymously, so that the results would not be biased.

This study was approved by the Ethical Committee of Getafe University Hospital.

### 2.1. Statistical Analysis

The sample size necessary to obtain significant differences with 80% of statistical power and an alpha error of 0.5 was calculated on the basis of previous studies which analysed NO levels in plasma in patients of similar condition [2, 3]. This sample size needed was estimated as 45 patients. Student's *t*-test was used for the end-points variables with normal distribution analysis and the Mann-Whitney *U*-test for those in which the distribution was not normal. The Kolmogorov-Smirnov and Shapiro-Wilk tests were used for the analysis of normality. The *χ* ^2^-test was used for categorical variables and the Spearman' *s*-test for the correlation between continuous variables. The data were expressed as mean ± standard deviation (SD) and the categorical as percentages. Statistical significance was assumed for *P* value <0.05.

## 3. Results

207 patients were consecutively assessed for inclusion in the study. Twenty-eight were excluded for not meeting inclusion criteria. Twenty-nine refused to participate, after we had explained in detail the conditions of the study and the hard recommendation of meeting the follow-up visits established per protocol. 150 patients with Fontaine stage II PAD were recruited and randomly assigned to each group, treatment (*n* = 75), and control (*n* = 75). The patient demographics features and current treatment are described in Table 1. Thus, there were no patient dropouts during the study. All 150 patients included completed the study protocol and were analysed for the primary outcome. No major adverse reactions to the treatment with statin were recorded.

**Figure 2:**
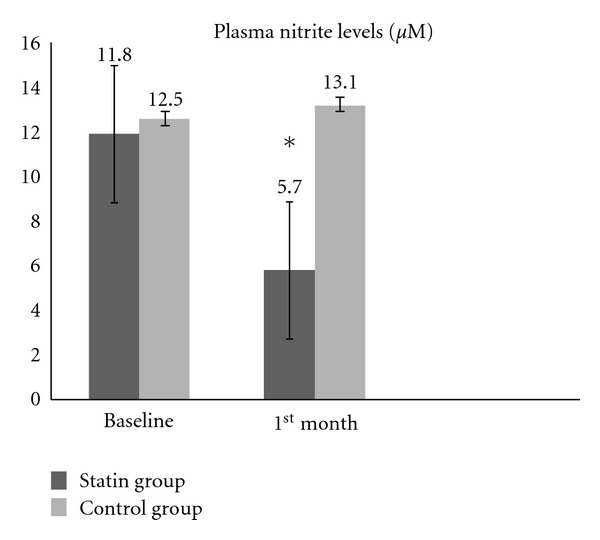
Comparison of plasma nitrite levels (*μ*M) at baseline and after a month of atorvastatin therapy (*P < 0.05 on comparison baseline versus a month treatment). There were no statistical differences between the control group data.

After one month of treatment with statin, a significant reduction in plasma nitrite levels was detected in treatment group patients (11.88 ± 7.8 *μ*M versus 5.7 ± 1.8 *μ*M, *P* < 0.0001) and in the comparison between treatment and control groups (5.7 ± 1.8 *μ*M versus 13.1 ± 9.1 *μ*M, resp., *P* < 0.001). No changes were found in the nitrite levels in the control group patients over the course of the study (12.5 ± 5.1 *μ*M versus 13.1 ± 9.1 *μ*M, *P* = 0.83) (Figure 2).

A significant reduction in plasma levels of total cholesterol, LDL cholesterol, total-cholesterol/HDL-cholesterol ratio, and total triglycerides was observed in treatment group (Table 2).

**Figure 3:**
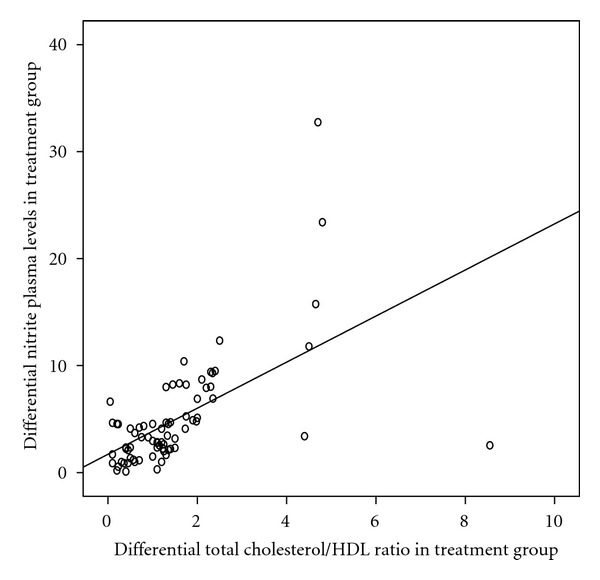
Positive Spearman's correlation between differential total cholesterol/HDL ratio and differential nitrite plasma levels in treatment group (*r* = 0.7, *P* < 0.001).

We defined differential of total-cholesterol/HDL-cholesterol ratio in treatment group as the difference between total-cholesterol/HDL-cholesterol ratio baseline value and total-cholesterol/HDL-cholesterol ratio 1st month treatment value. As well as differential of nitrite plasma levels in treatment group as the difference between nitrite basal levels and nitrite levels after one month of treatment with atorvastatin. A strong positive Spearman's correlation was found between the differential of nitrite levels in plasma and the differential of total-cholesterol/HDL-cholesterol ratio on atorvastatin group (*rs* 0.7; *P* < 0.001) (Figure 3).

## 4. Discussion

The current study evaluated and analysed in vivo, the effects of the HMG-coA reductase inhibitors upon variations of NO bioavailability and variations in plasma levels of total-cholesterol/HDL-cholesterol ratio, in patients with PAD. Our data revealed that decrease in total-cholesterol/HDL-cholesterol ratio was correlated with reduction of nitrite levels in patients with PAD and atorvastatin treatment.

Hypercholesterolemia is a well-established risk factor for atherosclerosis, in particular by decreasing the availability of nitric oxide [31]. Oxidative stress is known to contribute to atherogenesis, and, during this process, lipid peroxidation takes place in lipoproteins as well as in arterial macrophages [32]. The result is a drop in the bioavailability of endothelial NO, a reduction in the effective lumen of the vessel, and an increase in sensitivity to acetylcholine-induced vasoconstriction.

Several lines of evidence indicate that HDL has beneficial antiatherosclerotic effects [10, 11]. HDL acts as a reverse cholesterol transporter by removing excess cellular cholesterol to the liver, resulting in protection against atherosclerosis. This is the predominant antiatherosclerotic effect of HDL. However, HDL has multifactorial effects to protect vascular function. Thus, HDL activates endothelial NO synthase by enhancing Akt and MAP kinases leading to an increase in NO, suppresses endothelial cell apoptosis by activation of the Akt pathway and inhibition of caspase 3 and 9, inhibits the oxidation of LDLs, limits inflammation processes, stimulates prostacyclin production, and upregulates cyclooxygenase-2 expression leading to anticoagulation and vasodilation [10, 11]. Therefore, under a low HDL cholesterol condition, atherosclerotic processes develop progressively, resulting in cardiovascular impairment.

Previous studies related to the effects of atorvastatin therapy on lipid profile and oxidative stress revealed that atorvastatin therapy decreased the levels of oxidative stress [33]. An improvement on endothelial function has been described in patients diagnosed of PAD treated with atorvastatin at the beginning of the treatment. Nevertheless, this effect was not maintained at long term [34]. Different mechanisms by which atorvastatin improves endothelial function have been postulated. It is well known that statins augment endothelium-dependent vasodilation through an increase in NO bioavailability and decrease in oxidative stress and inflammation [35, 36]. In the present study, we have observed that four-week treatment with the HMG-CoA reductase inhibitor atorvastatin in patients with PAD decreased total-cholesterol/HDL-cholesterol ratio, and this is associated with the improvement on endothelial function provoked at the beginning of the statin treatment. According the results of this study, it could be hypothesized that a possible mechanism by which atorvastatin improves endothelial function is by its decreasing Total-cholesterol/HDL-cholesterol ratio effect. 

Nevertheless, recent studies have shown that atorvastatin has more beneficial effects on endothelial function than other statins at similar lipid reduction [37]. However, the conclusions from other studies remain inconsistent [38] contributing to certain controversy.

Anyway, Sugiura et al. have previously described an inverse correlation between total-cholesterol/HDL-cholesterol ratio and endothelial function by flow-mediated dilatation [39]. Our data support their results through variations of nitrite plasma levels. In addition, these findings underline the importance of plasma total-cholesterol/HDL-cholesterol values as a risk factor of endothelial dysfunction in PAD patients. It is noteworthy that the correlation between nitrite levels and total-cholesterol/HDL-cholesterol found in the current study was mainly determined by increasing levels of HDL after atorvastatin treatment.

The limitations of this study include the fact that nitrite levels are influenced by endogenous and exogenous factors such as dietary nitrates, the inhalation of atmospheric NO, its formation in the salivary glands, and renal function. Although it is not possible to exclude these factors, these data are in line with observations from earlier studies. While dealing with plasma nitrites and not other nitrates may seem to be a limitation, it is known that over 70%–90% of all plasma nitrites originate from eNOS activity [40].

## 5. Conclusions

The present study demonstrated that improvement of nitrite plasma levels, a marker of endothelial function, is associated with decreased total cholesterol/HDL ratio values in patients with PAD treated with atorvastatin. In the early stages of PAD, the impact of total-cholesterol/HDL-cholesterol ratio may relate to endothelial damage.

## Figures and Tables

**Table 1:** Demographic data and treatment.

	Treatment group	Control group	*P*
	*N* (75) (%)	*N* (75) (%)	
Age	71.4 ± 10.8	70.51 ± 9.7	n.s
Male	65 (87)	62 (82.6)	n.s
Hypertension	55 (73.3)	57 (76)	n.s
DM	30 (40)	33 (44)	n.s
Current smoking	41 (55)	39 (52)	n.s
Ex smoker	26 (35)	32 (42.6)	n.s
AMI	10 (14)	8 (10.6)	n.s
Dyslipidaemia	18 (25)	12 (16)	n.s
CVA	5 (6.6)	6 (8)	n.s
Antiplatelet treatment	65 (86.6)	69 (92)	n.s
ACE inhibitors	35 (46.7)	30 (40)	n.s
ARA-II	10 (13.3)	8 (10.6)	n.s
B-blocker	11 (14.6)	12 (16)	n.s
Nitrites	5 (6.7)	3 (4)	n.s
CA antagonists	15 (20)	17 (22.6)	n.s

DM: diabetes mellitus; AMI: acute myocardial infarction; CVA: cerebrovascular accident.

**Table 2:** Initial lipid-profile values and values after 1 month of treatment with atorvastatin.

	Mean ± DS	*P*
Total CHOL baseline	5.4 ± 1.3 mmol L^−1^	0.0001
Total CHOL after treatment	3.8 ± 0.1 mmol L^−1^	
		
LDL-CHOL baseline	3.4 ± 1. mmol L^−1^	0.0001
LDL-CHOL after treatment	1.8 ± 0.6 mmol L^−1^	
		
HDL-CHOL baseline	1.3 ± 0.3 mmol L^−1^	0.12
HDL-CHOL after treatment	1.4 ± 0.4 mmol L^−1^	
		
CHOL/HDL baseline	4 ± 0.9 mmol L^−1^	0.0001
CHOL/HDL after treatment	2.7 ± 0.6 mmol L^−1^	
		
TGC baseline	1.3 ± 0.5 mmol L^−1^	0.024
TGC after treatment	1.1 ± 0.4 mmol L^−1^	
		
TGC/HDL baseline	2.3 ± 1.3 mmol L^−1^	0.113
TGC/HDL after treatment	2 ± 1.2 mmol L^−1^	
